# Bibliographical

**Published:** 1872-08

**Authors:** 


### BIBLIOGRAPHICAL.

*Lectures on the Priniciples and Practice of Physic.* Delivered at King's College, London, by Sir Thomas Watson, Bart., M.D., F.R.S., Physician in Ordinary to the Queen, etc., etc. In Two Volumes. From the Fifth Revised and Enlarged English Edition. Edited, with Additions and Numerous Illustrations, by Henry Hartshorne, A.M., M.D., Professor of Hygiene in the University of Pennsylvania. Henry C. Lea, Publisher, Philadelphia.

It is with real pleasure that we again open the pages of Watson, brought up in a fifth edition, by competent hands, to the present status of medical science.

As remarked by the more recent editor (Dr. Condie having edited an edition in 1858), since the time of the first publication of these lectures, they have been, in some respects, without a rival—not as an exhaustive treatise (which was not the author's design), but as presenting the most lucid and interesting exposition of the practice of medicine, in the light of careful research and large experience, which our language affords. The justification which the editor feels to be needful for venturing to make occasional additions, is found in some of the words of the author's epilogue, at the close of his book—that "A few things in the first of these volumes would have been differently put, if they had not been already printed while I was preparing the second." The different aspect, moreover, of some diseases upon this side of the ocean, and the greater relative importance of certain topics, make suitable the introduction of a few additional pages for American readers.

Whenever it could be done with justice to the present state of medical opinion and experience, Dr. Condie's notes and additions, made in the edition of 1858, have been used. The most extended articles by Dr. Condie are those upon remittent fever, yellow fever, cholera infantum, and cerebro-spinal meningitis. The present editor (Dr. Hartshorne) has commented at the greatest length upon medical thermometry, the pathology of croup, the causation and prevention of yellow fever and cholera. Numerous wood-cut illustrations have been introduced throughout the work, as in the previous American edition. The text of the author has not been altered, the editorial additions being by brackets or an initial. It should be in the hands of every student and in the library of every practitioner.

*On Food: Its Varieties, Nutritive Value, Comparative Digestibility, Physiological Functions and Uses, Preparation, Culinary Treatment, etc.* Being the Substance of Four Cantor Lectures, by H. Letheby, M.B., M.A., etc.

The writer of this work is Professor of Chemistry in the College of the London Hospital, and Medical Officer of Health and Food Analyst for the City of London. In a neat volume of two hundred and forty-six pages, he discusses the various subjects embraced in the above comprehensive title in a concise, yet clear and attractive style. While no effort is made at scientific and technical display, the writer exhibits a minute and thorough acquaintance with the science of his subject in all its departments, physiological, historical, economic and hygienic. This is seen in numerous dietary tables for prisons, hospitals, etc.; in the attempt to fix the labor value of different kinds of food, or the kind which will give the greatest muscular and vital force, with the least expense; in his critical review of Liebig's chemico-physiological views; and, finally, in his extensive knowledge of the dietetic habits of apparently every nation, savage and civilized.

The work is a most valuable one, and should be read by every physician. If we would maintain the honor of our profession; if we would achieve individual success; if we aspire to be more than drug-mongers and empirics; we must keep pace with the progress of science by studying such works as these. We must learn that the materia alimentaria has no less to do with the cure of disease than the materia medica. Then will a physician no longer be considered as having discharged all his obligations when he prescribes his pills, potions and powders, and takes his departure without a word of direction as to what his patients shall eat or drink.

The work, which merits a more extended notice, is published by the well-known publishers, Wm. Wood & Co., New-York.

Other books and publications (among them, "Gross' New Surgery") will be noticed in the next number of the Journal.

